# Consensus statement addressing controversies and guidelines on pediatric urolithiasis

**DOI:** 10.1007/s00345-024-05161-4

**Published:** 2024-08-07

**Authors:** S. Güven, T. Tokas, A. Tozsin, B. Haid, T. S. Lendvay, S. Silay, V. C. Mohan, J. R. Cansino, S. Saulat, M. Straub, A. Bujons Tur, B. Akgül, J. Samotyjek, L. Lusuardi, S. Ferretti, O. F. Cavdar, G. Ortner, S. Sultan, S. Choong, S. Micali, I. Saltirov, A. Sezer, C. Netsch, E. de Lorenzis, O. O. Cakir, G. Zeng, A. S. Gozen, G. Bianchi, B. Jurkiewicz, T. Knoll, J. Rassweiler, K. Ahmed, K. Sarica

**Affiliations:** 1 Department of Urology, Necmettin Erbakan University Meram School of Medicine, Konya, Turkey; 2 Department of Urology, University General Hospital of Heraklion, Athens, Greece; 3 Department of Urology, Trakya University School of Medicine Hospital, Edirne, Turkey; 4 Ordensklinikum Linz, Barmherzige Scwestern Hospital, Linz, Austria; 5 Department of Urology, University of Washington, Seattle Children’s Hospital, Seattle, WA USA; 6 Istanbul Medipol University, Istanbul, Turkey; 7 Preeti Urology Hospital, Hyderabad, Telangana India; 8 Hospital Universitario La Paz, Madrid, Spain; 9 Department of Urology, Tabba Kidney Institute, Karachi, Pakistan; 10 Department of Urology, Technical University Munich, Munich, Germany; 11 Urology Department, Fundación Puigvert, Universidad Autónoma de Barcelona, Barcelona, Spain; 12 Pediatric Surgery and Urology Clinic CMKP in Dziekanów Leśny, Dziekanów Leśny, Poland; 13 Department of Urology, Paracelsus Medical University Salzburg University Hospital, Urology, Salzburg, Austria; 14 Department of Urology, University of Modena and Reggio Emilia, Modena, Italy; 15 Department of Urology, General Hospital Hall I.T, Tirol, Austria; 16 Department of Urology, Menoufia University Hospitals, Shebeen El Kom, Egypt; 17 Institute of Urology, University College Hospital, London, UK; 18 Department of Urology and Nephrology at Military Medical Academy, Sofia, Bulgaria; 19 Pediatric Urology Clinic, Konya City Hospital, Konya, Turkey; 20 Asklepios Klinik BarmbekAbteilung Für Urologie, Hamburg, Germany; 21 Department of Urology, Fondazione IRCCS Ca’ Granda Ospedale Maggiore Policlinico, Milan, Italy; 22 King’s College London, Guy’s and St. Thomas’ NHS Foundation Trust, King’s Health Partners, London, UK; 23 Department of Urology and Guangdong Key Laboratory of Urology, The First Affiliated Hospital of Guangzhou Medical University, Guangzhou, China; 24 Department of Urology, Medius Clinic, Ostfildern, Germany; 25 Klinikum Sindelfingen-Boeblingen, Sindelfingen, Germany; 26 Department of Urology and Andrology, Danube Private University, Krems, Austria; 27 Sheikh Khalifa Medical City, Abu Dhabi, UAE; 28 Khalifa University, Abu Dhabi, UAE; 29 Sancaktepe Sehit Prof. Dr. Ilhan Varank Research and Training Hospital, Istanbul, Turkey; 30 Department of Urology, Biruni University Medical School, Istanbul, Turkey

**Keywords:** Pediatric urolithiasis, ESWL, PCNL, Ureteroscopy, Metabolic evaluation, Guidelines

## Abstract

**Purpose:**

We aimed to investigate controversial pediatric urolithiasis issues systematically, integrating expert consensus and comprehensive guidelines reviews.

**Methods:**

Two semi-structured online focus group meetings were conducted to discuss the study’s need and content, review current literature, and prepare the initial survey. Data were collected through surveys and focus group discussions. Existing guidelines were reviewed, and a second survey was conducted using the Delphi method to validate findings and facilitate consensus. The primary outcome measures investigated controversial issues, integrating expert consensus and guideline reviews.

**Results:**

Experts from 15 countries participated, including 20 with 16+ years of experience, 2 with 11–15 years, and 4 with 6–10 years. The initial survey identified nine main themes, emphasizing the need for standardized diagnostic and treatment protocols and tailored treatments. Inter-rater reliability was high, with controversies in treatment approaches (score 4.6, 92% agreement), follow-up protocols (score 4.8, 100% agreement), and diagnostic criteria (score 4.6, 92% agreement). The second survey underscored the critical need for consensus on identification, diagnostic criteria (score 4.6, 92% agreement), and standardized follow-up protocols (score 4.8, 100% agreement).

**Conclusion:**

The importance of personalized treatment in pediatric urolithiasis is clear. Prioritizing low-radiation diagnostic tools, effectively managing residual stone fragments, and standardized follow-up protocols are crucial for improving patient outcomes. Integrating new technologies while ensuring safety and reliability is also essential. Harmonizing guidelines across regions can provide consistent and effective management. Future efforts should focus on collaborative research, specialized training, and the integration of new technologies in treatment protocols.

**Supplementary Information:**

The online version contains supplementary material available at 10.1007/s00345-024-05161-4.

## Introduction

The incidence of pediatric urolithiasis is increasing, contributing to significant clinical challenges and growing medical costs. Risk factors for pediatric urolithiasis are multifactorial and include regional, racial, gender, socioeconomic, and dietary variations [1–4]. Although technological advances have led to breakthroughs in the surgical treatment of urolithiasis, faster supporting clinical evidence is required to keep up with these technological and industrial advances [5, 6]. Furthermore, our profession demands more progress in understanding the pathogenesis, preventive measures, and medical treatment of stone disease.

Innovations initially applied to adult patients have been adapted for pediatric use, often with minimal modifications [7–9]. Although the approach and interventional techniques for treating stone disease in children are similar to those in adults, pediatric stone patients differ from adults in many ways. The direct application of adult techniques to children sometimes needs to account for these populations’ unique anatomical and physiological differences in these populations. The assumption that adult-derived guidelines and experiences entirely apply to pediatric patients is flawed.

Computer science and technological advancements have facilitated data collection for subsequent imaging modalities, stone surveillance, and the management of stones through minimally invasive and endoscopic techniques [10, 11]. These advancements have created opportunities to develop new, productive, high-standard clinical trials [12]. Nevertheless, the consensus is lacking on the appropriate evaluation methods. It is imperative to systematically compile and organize long-standing and emerging contentious issues in pediatric urolithiasis to address the challenges of this new era. Although current guidelines are prepared at the highest level of evidence and are readily available, to keep pace with the rapid advancements in science, technology, and information, it is necessary to standardize the different pediatric urolithiasis guidelines and recommendations, each developed and continuously updated by experts with great effort but in different styles, through a structured approach [13, 14].

In this study, we collaborated with experts and residents to investigate controversial issues using a systematic approach, integrating expert viewpoints and comprehensive reviews of guidelines to address the controversies.

## Methods

### Type of study/location

This study used a combination of qualitative and quantitative methodologies and involved 26 adult and pediatric urologists and four residents from the European Association of Urology Working Group on Paediatric Urology (EWPU), the European Association of Urology Section of Urotechnology (ESUT), the European Association of Urology Section of Urolithiasis (EULIS), and the International Alliance of Urolithiasis (IAU).

### Development phase

**Fig. 1:**
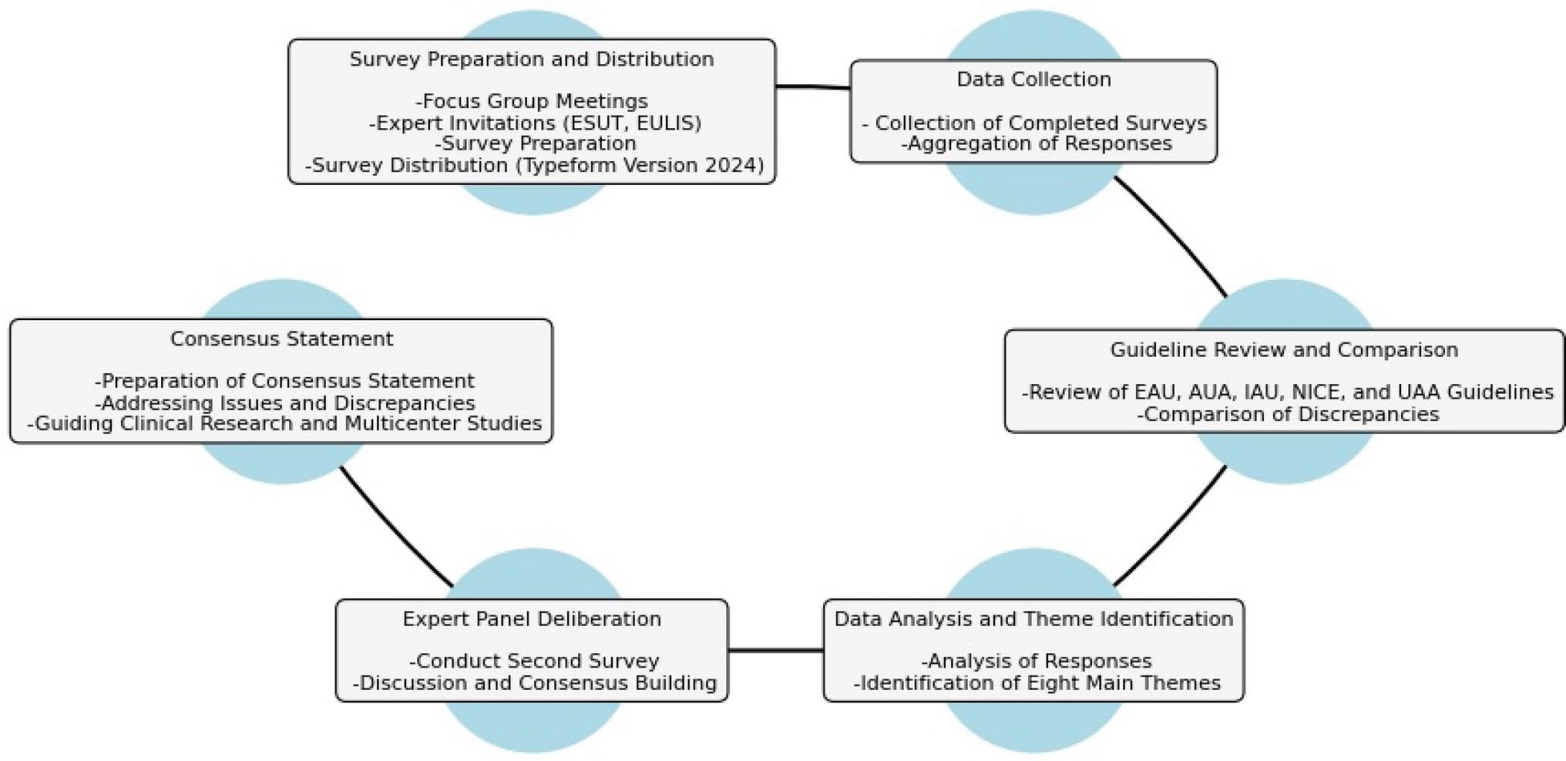
Flowchart of the study methodology

The study methodology was developed based on insights from expert opinions, focus group discussions, and comprehensive literature reviews and comprised several structured phases, including survey preparation and distribution (Fig. 1). SG, KA, AT, and BA conducted two separate semi-structured focus group online meetings. The first focus group discussed the need and content of the study, during which current literature was reviewed to investigate controversial issues and provide up-to-date information. Based on this review, the initial survey developed via Typeform (Version 2024, Typeform, Inc.) (Supplement 1) was distributed to invited experts.

### Data collection and analysis

Once the first round was completed, the data collection phase involved aggregating all responses. The responses were then analyzed to identify key issues and patterns. This analysis identified nine main themes that encapsulate the core controversial issues in pediatric urolithiasis.

### Guideline reviews

Following the data analysis, a comprehensive review of existing guidelines was undertaken. A comprehensive web search for urolithiasis guidelines was performed using the keywords: "pediatric urolithiasis," "urolithiasis," and "guidelines." This search aimed to identify guidelines specifically addressing urolithiasis in children. Six relevant guidelines were identified [15–25]. The included guidelines were the European Association of Urology (EAU) Pediatric Urology Guidelines [15], the EAU Urolithiasis Guidelines [16], the American Urological Association (AUA) Kidney Stone Guidelines [17, 18], and the International Alliance Urolithiasis (IAU) Guidelines [19–23], the Urological Association of Asia (UAA) clinical guideline for urinary stone disease [24], National Institute for Health and Care Excellence (NICE): Guidelines [25]. The recommendations and the stated degrees of supporting evidence in these guidelines were independently evaluated by three authors (OFC, AT, BA). Guideline statements were then correlated according to controversial issues, and a comparative analysis was conducted to determine areas of consensus and conflict. Potential research priorities were identified based on the information obtained from the first round—an initial opinion survey—and a thorough examination of the guidelines.

### Validation phase

A second round was conducted among the same group of experts (Supplement 2) to elaborate further and validate the findings. This survey focused on discussing the findings from the initial analysis and guideline review. Its aim was to facilitate consensus on the controversial issues and integrate a comparison of the guidelines. Finally, a consensus statement was prepared to address the identified issues and guideline discrepancies, highlighting research priorities. The consensus statement guides clinical research and multicentric studies, providing a cohesive direction for future investigations into pediatric urolithiasis.

### Delphi process

The Delphi process involved two rounds of structured surveys to gather and refine expert opinions systematically. In the first round, an initial survey was developed based on a literature review and insights from focus group discussions. The second round surveyed the same group of experts to further elaborate and validate the findings from the first round. A 5-point Likert scale was used with the determined subheadings. The process aimed to achieve a high level of consensus on identified issues, with a total acceptance percentage of 95%. The final consensus statement was prepared to address the identified issues and guideline discrepancies, highlighting research priorities and providing direction for future investigations into pediatric urolithiasis.

Figures were created using Python (Version 3.12.3).

## Results

### Development of quantitative survey

#### First round of Delphi

**Fig. 2:**
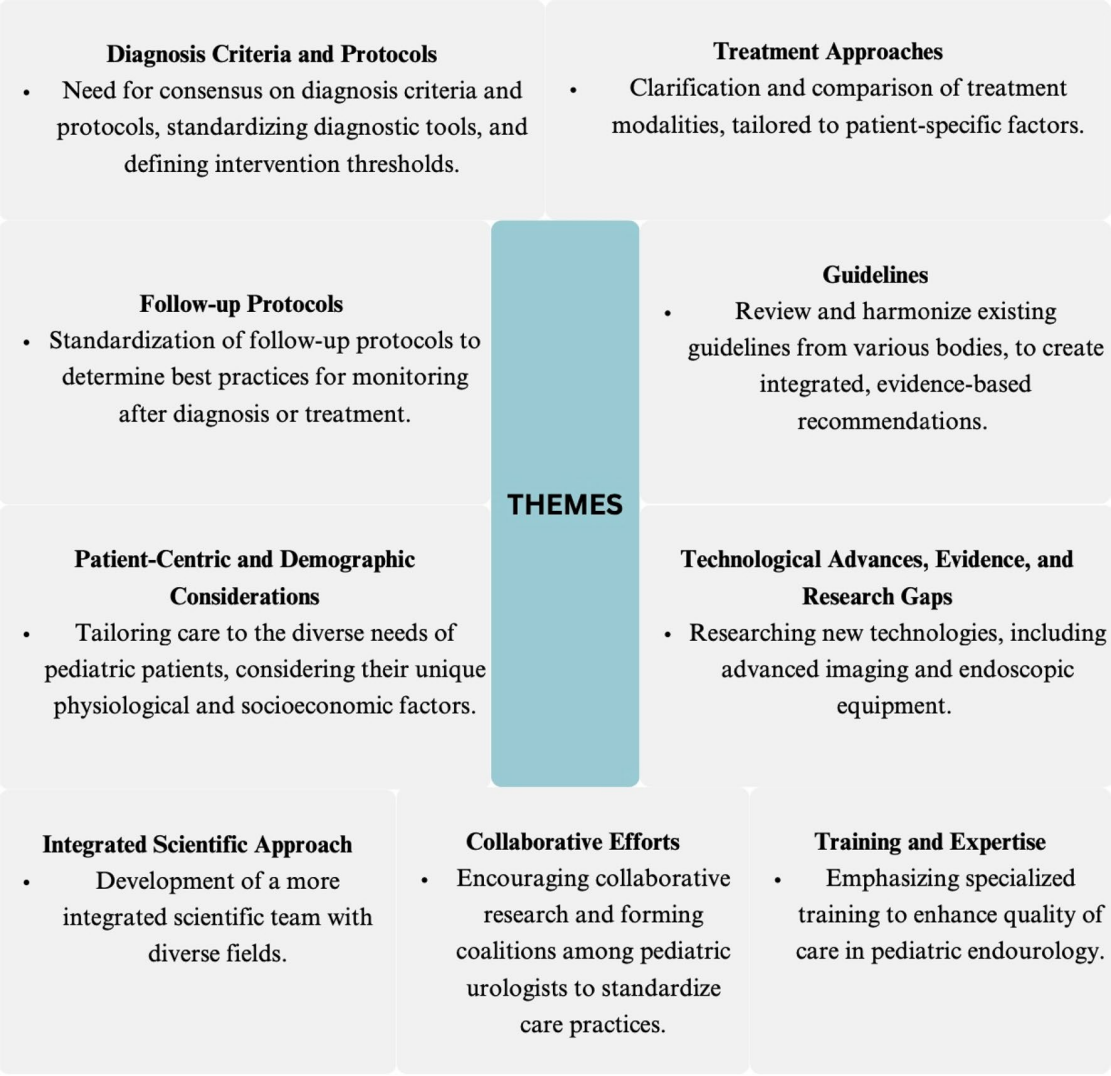
Responses from the initial survey and the nine main themes

The initial survey for the Pediatric Urolithiasis Consensus Study gathered responses from experts from 15 countries with varying years of experience: 20 had 16+ years, two had 11–15 years, and four had 6–10 years. The responses were aggregated and analyzed to identify significant themes, identifying nine main themes (Fig. 2). The inter-rater reliability in the development of themes was high, with the primary areas of controversy being optimal treatment approaches (average score of 4.6, 92% agreement), variability in follow-up protocols (average score of 4.8, 100% agreement), and the lack of consensus on diagnostic criteria (average score of 4.6, 92% agreement). The primary factors contributing to the controversies included variability in diagnostic and therapeutic protocols, the lack of standardized guidelines tailored specifically for pediatric patients, and differing levels of expertise and experience among urologists. Overall, the rigorous data collection, analysis, and theme identification processes were praised, receiving high scores with average points around 4.6 and agreement at 100%. The expert panel deliberation methodology and the drafting of the consensus statement were also highly regarded, with average scores of 4.7 to 4.8 and agreement at 96 to 100%.

### Review of guidelines

The guidelines reviewed included the EAU Pediatric Urology Guidelines [15], the EAU Urolithiasis Guidelines [16], the AUA Kidney Stone Guidelines [17] and the IAU guidelines [19–23], the Urological Association of Asia (UAA) clinical guideline for urinary stone disease [24], National Institute for Health and Care Excellence (NICE): Guidelines [25]. A comprehensive comparative overview of the disparities in recommendations for pediatric urolithiasis is presented in Table 1. Each guideline offered appropriate recommendations based on the latest evidence, and consensus was achieved in many areas. However, discrepancies were observed, particularly in the preferred diagnostic tools, treatment methods, and follow-up protocols. The EAU Urolithiasis Guidelines recommend SWL for kidney stones measuring 10–20 mm, and the EAU Pediatric Urology Guidelines suggest using SWL for upper ureteral stones, with the possibility of needing flexible scopes in case of retropulsion. Also, in cases of renal pelvis stones measuring 10–20 mm, SWL, RIRS and PCNL have similar recommendation grades according to the EAU Pediatric Urology guidelines. The AUA offers SWL or URS based on stone burden and anatomy, and the IAU considers SWL the first line for staghorn calculi in non-dilated systems. For stones larger than 10 mm, the EAU Urolithiasis Guidelines recommend URS. On the other hand, Pediatric urology guidelines recommend PCNL as the primary option for lower pole stones >10 mm. The EAU Pediatric Urology Guidelines suggest URS for lower ureteral stones, with SWL as an alternative, whereas the AUA favors URS over SWL for ureteral stones needing intervention. The EAU Urolithiasis Guidelines advise PCNL for stones over 20 mm or in complex cases. The EAU Pediatric Urology also recommends PCNL for staghorn stones. The AUA recommends SWL as the first-line treatment for staghorn calculi in non-dilated collecting systems, while PCNL is considered acceptable for renal stones larger than 20 mm. The EAU Pediatric Urology Guidelines advocate age-tailored management, recommending tailored approaches for pediatric patients and avoiding open surgery when possible. The AUA recommends active surveillance with ultrasonography for asymptomatic and non-obstructing renal stones [18–20].

**Table 1:** Comparative overview of differences in pediatric urolithiasis recommendations in the guidelines

Aspect	EAU urolithiasis guidelines	EAU pediatric urology guidelines	AUA guidelines	IAU guidelines	NICE guidelines	UAA guidelines
Diagnostic Approach	USG and KUB as first-line; non-contrast CT for details	USG typically first approach (plain abdominal X-ray); non-contrast helical CT when more sensitive testing is needed	Low-dose CT scan for pediatric patients prior to PCNL; initial ultrasound assessment emphasized	Not specified	USG as first line; low-grade non-contrast CT	USG as first line; low-grade non-contrast CT
Stone composition	Emphasizes calcium oxalate stones	Over 70% of stones in children contain calcium oxalate; younger children may have infectious stones	Not specified in the provided text	Not specified	Not specified	Not specified
Management for small stones	Expectant management for stones <10 mm	Not specified	Observation or MET for uncomplicated ureteral stones ≤10 mm	Not specified	Not specified	Observation or MET for uncomplicated ureteral stones ≤10 mm
Shockwave lithotripsy (SWL)	Recommended for stones 10–20 mm	Recommended for upper ureteral stones; may use flexible scopes for retropulsion	SWL or URS offered based on stone burden and anatomy	Considered first-line for staghorn calculi in non-dilated collecting systems	SWL for stones <10 mm	Not specified
Ureteroscopy (URS)	URS for stones >10 mm	URS for lower ureteral stones; SWL as an alternative	URS recommended over SWL for patients with ureteral stones needing intervention	Not specified	Recommended for stones 10–20 mm	Not specified
Percutaneous nephrolithotomy (PCNL)	PCNL for stones >20 mm or complex cases	PCNL recommended for staghorn stones; open/SWL as alternatives	PCNL as an acceptable option for renal stone burden >20 mm	Not specified	PCNL for stones >20 mm including staghorn stones	Not specified
Metabolic evaluation	Emphasized for prevention; MET can reduce expulsion time but may have side effects	Not specified	Metabolic testing suggested for high-risk or recurrent stone formers	Not specified	Consider referral to a pediatric nephrologist or pediatric urologist who specializes in this field	Metabolic evaluation including 24-h urine collection is recommended
Minimally invasive techniques	Prefers minimally invasive techniques when possible	Not specified	Encourages minimally invasive techniques with considerations of benefits and risks	Not specified	Not specified	Not specified
Age-tailored management	Not specified	Tailored approaches for pediatric patients; avoid open surgery when possible	Active surveillance with ultrasonography for asymptomatic and non-obstructing renal stones	Not specified	Not specified	Not specified

### Research priorities and areas for further study

The initial opinion survey and thorough examination of guidelines have identified ten potential research areas in pediatric urolithiasis (Table 2). These research priorities aim to enhance diagnostic accuracy, standardize treatment protocols, address the need for pediatric-specific evidence, explore socioeconomic and institutional factors, emphasize specialized training, promote global standardization, understand disease mechanisms and genetic causes, advance endoscopic technologies, and investigate the long-term effects of treatments (Table 2).

**Table 2:** Research priorities in pediatric urolithiasis

Research priorities and areas for further study	Description
Diagnostic criteria and tools	• Enhanced research is necessary to decrease variability and controversies in diagnostic criteria and to develop pediatric-specific diagnostic tools and algorithms • There is a call for establishing evidence for a clear diagnosis algorithm, and more studies to define stone size in relation to patient demographics, emphasizing that a stone in an infant does not equate to one of the same size in an adolescent
Treatment modalities and approaches	• Considering stone size, location, and patient symptoms • Research into the role and duration of metaphylaxis, the treatment decision-making process, and the timing for DJ stent removal is also needed • There is a need for research into the use of miniaturized scopes, such as 4.9 F or 6.5 F flexible URS, in pediatric patients due to high pre-stenting rates with adult-sized instruments • The evaluation of emerging technologies should assess both their clinical efficacy and the environmental impact of the disposable components used • The most effective treatment methods, including different techniques and interventions in various patient positions (supine/prone)
Monitoring and follow-up care	• Prioritizing the standardization of follow-up protocols • Reducing radiation exposure from imaging techniques • Managing residual fragments • Developing strategies for long-term monitoring • Preventing recurrence.
Evidence and guidelines	• Addressing the shortage of pediatric-specific evidence is crucial for developing standardized guidelines • While current guidelines offer the best available evidence, their recommendation grades are often low due to evidence scarcity • There is a need for high-quality evidence to enhance these recommendations • In the interim, it is vital to tailor decisions to individual patient circumstances
Socioeconomic and institutional factors	• Research should investigate the influence of socioeconomic conditions on management • The variability due to institutional protocols, and access to care and treatment technologies
Professional training and specialization	• The importance of specialized training in pediatric endourology and the necessity for dedicated pediatric urologists are emphasized • Different techniques and interventions in various patient positions (supine/prone) • Professional development in the latest techniques and technologies is also a significant research priority
Collaborative efforts and global perspectives	• Efforts towards consensus guidelines and global standardization must continue, alongside collaborative research projects and the inclusion of diverse healthcare settings and populations
Bench to bedside scientific and informatic advancements	• Pathogenesis, Prevention, and Treatment Studying mechanisms, prevention, and treatments for kidney stones • Genetic Causes and Sequencing • Investigating mRNA-based therapies • Exploring the microbiome's role in stone risk • Applying informatics and AI from pathogenesis and diagnosis to follow-up in urolithiasis
Treatment equipment and innovation	• Further research is necessary for the miniaturization of surgical instruments, especially the development and use of scopes suitable for pediatric patients • Advancements in endoscopic technologies, suction devices, new-generation lasers • The exploration of the critical role of AI in diagnosis and treatment
Long-term impact and comparative studies	• Long-term effects of SWL on kidney function and hypertension • The thermal effects of lasers in the kidney and the ureter • Multicentric RCTs comparing PNL, RIRS, and SWL • The continued research into medications for the prevention of recurrence

### Second round of Delphi

In the second round of the survey, a 5-point Likert scale was used to assess the experts’ opinions (Supplement). A critical need for consensus on diagnosis criteria and protocols was underscored, with an average score of 4.6 and 92% agreement. It was unanimously agreed that standardizing diagnostic tools and defining clear intervention thresholds are essential, receiving an average score of 4.6. The need for clarifying and comparing treatment methods tailored to patient-specific factors was prioritized, with an average score of 4.6 and 92% agreement. Additionally, there was strong support for standardizing follow-up protocols to establish best practices, achieving the highest average score of 4.8 with 100% agreement.

Addressing the lack of pediatric-specific evidence and research to guide clinical decisions was identified as urgent, reflected by an average score of 4.6 and full agreement. Similarly, the need to review and harmonize existing guidelines and create integrated, evidence-based recommendations garnered unanimous support with an average score of 4.6. Enhancing specialized training in pediatric endourology was considered vital, with an average score of 4.6 and 92% agreement. The emphasis on research into new technologies, including advanced imaging and endoscopic equipment, was acknowledged, though with slightly lower agreement, indicated by an average score of 4.5 and 88% agreement.

The survey highlighted the importance of tailoring pediatric urolithiasis care to diverse patient needs was highlighted by the survey, achieving an average score of 4.6 and 96% agreement. Encouraging collaborative research and the formation of coalitions among pediatric urologists was also emphasized, with an average score of 4.5 and 92% agreement. The comparative analysis of guidelines revealed important discrepancies and areas needing alignment, particularly in diagnostic approaches, treatment modalities for small stones, and recommendations for procedures like shockwave lithotripsy, ureteroscopy, and percutaneous nephrolithotomy, with average scores ranging from 4.1 to 4.6 and agreement percentages between 76 and 100%.

The rigorous data collection, analysis, and theme identification processes were praised, receiving high scores with average points around 4.6 and agreement at 100%. The expert panel deliberation methodology and the drafting of the consensus statement were also highly regarded, with average scores of 4.7–4.8 and agreement at 96–100%.

## Discussion

In the last few decades, there has been significant progress in the treating urolithiasis, driven by the enthusiasm of urologists and industry focusing on minimally invasive techniques. However, despite international best practice guidelines, insufficient evidence exists specific to pediatric urolithiasis [26, 27]. The literature often needs robust, high-quality studies that address pediatric patients’ unique needs across all management stages, from diagnosis and metabolic evaluation to treatment and follow-up. As a result, individualized patient approaches often influence clinical decision-making, the clinical experience of the urologist, and regional urology practices. Key differences must be recognized in several areas. Diagnosis and metabolic evaluation are crucial due to the high risk of recurrence in pediatric patients, necessitating thorough metabolic assessments to identify underlying disorders predisposing children to stone formation. Imaging in pediatric patients must minimize radiation exposure due to their increased vulnerability and the long-term risk of oncological conditions, requiring using low-radiation or radiation-free modalities whenever possible [28–30].

The consensus among experienced urologists is critical in managing pediatric urolithiasis. In this consensus study, our goal was to highlight the ongoing discussions and unresolved issues despite the extensive and productive literature available. Experts generally perceived current practices and recommendations regarding pediatric urolithiasis management as highly variable, often influenced by adult treatment protocols and lacking specific pediatric-focused evidence. Key areas identified for further research included decision-making processes in management and follow-up of pediatric cases, the development and standardization of diagnostic tools and treatment protocols, and the role of metabolic evaluation and medical prevention in improving patient outcomes. Further research and high-quality evidence are necessary to enhance the diagnosis, treatment, and long-term management of pediatric patients with urolithiasis.

Our consensus study on pediatric urolithiasis revealed a strong agreement among the experts on several critical areas. Most emphasized establishing standardized diagnostic criteria and protocols, including clear intervention thresholds and uniform diagnostic tools. There was significant consensus on tailoring treatment approaches to individual patient factors and ensuring that treatments are compared to make personalized treatment decisions.

Medical management must be tailored to children’s unique pharmacokinetics and pharmacodynamics, ensuring appropriate dosing and safety profiles [31]. Interventional procedures, such as extracorporeal shock wave lithotripsy (ESWL) and other surgical interventions, must be cautiously approached to avoid disrupting the developing renal parenchyma and affecting kidney growth and function [32–34]. Post-treatment management is critical, as pediatric patients are at a higher risk of recurrence and may have residual stone fragments, necessitating long-term follow-up to monitor renal function and prevent recurrent stone formation [35].

The need for standardized follow-up protocols was highlighted to establish best practices for monitoring children post-diagnosis or treatment. Experts emphasized the lack of pediatric-specific evidence and research on residual fragments, a longstanding challenge for urologists, crucial for guiding clinical decisions and improving patient outcomes. Many patients, especially those with staghorn stones, have residual fragments after treatment. No consensus on the imaging modality or timing for evaluating these fragments [35, 36]. Treatment options for residual stones vary based on the department’s facilities and the surgeon’s preference, including active surveillance, SWL, RIRS, or a second look PNL [36].

Comparing guidelines reveals notable differences and areas of consensus. For diagnostic approaches, the EAU Urolithiasis Guidelines recommend USG and KUB as first-line tools for diagnostic approaches, with non-contrast CT for detailed imaging [16]. The EAU Pediatric Urology Guidelines similarly advocate for USG first, supplemented by non-contrast helical CT when needed [15]. The AUA Guidelines emphasize low-dose CT scans for pediatric patients before PCNL, with initial ultrasound assessments [17]. Regarding stone composition, the EAU guidelines emphasize calcium oxalate stones, aligning with the EAU Pediatric Urology Guidelines, which indicate over 70% of stones in children contain calcium oxalate. For managing small stones, the EAU Urolithiasis Guidelines suggest expectant management for stones under 10 mm for managing small stones.

In comparison, the AUA recommends observation or medical expulsive therapy (MET) for ureteral stones 10 mm or less. The guidelines from various organizations need to be reviewed and aligned to identify their compatibility with each other, as well as any inconsistencies and areas that are lacking concerning pediatric urolithiasis. It is important to prioritize the development of integrated, evidence-based recommendations. The need for specialized training and research in new technologies, such as advanced imaging and endoscopic equipment, was also emphasized to improve the quality of care in pediatric endourology. Collaborative research efforts and the formation of working groups among pediatric urologists were recognized as vital for standardizing pediatric urolithiasis care. Research into the safety and reliability of new-generation lasers, flexible and navigable ureteral access sheaths, and suction devices is necessary [37, 38]. The consensus results reflect a strongly align on these priorities, indicating a collective movement towards improving the diagnosis, treatment, and management of pediatric urolithiasis.

The study has a few limitations. Using expert opinion and survey-based data collection may introduce bias since these methods rely on the subjective views of the participants. Additionally, the study mainly involved experts from European and international urology associations, which may need to represent regional variations in practice and guidelines. The focus groups and surveys were conducted online, which might have limited the depth of discussions. Lastly, while the study identified key controversial issues and research priorities, it is limited by the need for robust pediatric-specific evidence in the current literature.

Our consensus study has identified critical areas of agreement and controversy in the managing pediatric urolithiasis. The existing guidelines are well-prepared in terms of best practices. However, the study emphasizes the importance of individualized treatment approaches, newly introduced equipment, and consideration of patient-specific factors to improve outcomes. In today’s fast-paced world of information and experiences, the existing levels of accumulated knowledge and evidence need help to keep pace with rapid advances in science, technology, and informatics, particularly in clinical practice. Moreover, our findings highlight that harmonizing guidelines across different organizations and regions can be beneficial to ensure consistent and effective management for pediatric urolithiasis patients. Future efforts should prioritize collaborative research, specialized training, and the integration of new technologies to enhance care quality and improve long-term outcomes for these vulnerable pediatric urolithiasis cases.

## Supplementary Information

Below is the link to the electronic supplementary material.

Supplementary file1 (DOCX 17 KB)

Supplementary file2 (DOCX 87 KB)

## Data Availability

No datasets were generated or analysed during the current study.
