# Correction: Consensus statement addressing controversies and guidelines on pediatric urolithiasis

**DOI:** 10.1007/s00345-024-05282-w

**Published:** 2024-09-30

**Authors:** S. Güven, T. Tokas, A. Tozsin, B. Haid, T. S. Lendvay, S. Silay, V. C. Mohan, J. R. Cansino, S. Saulat, M. Straub, A. Bujons Tur, B. Akgül, J. Samotyjek, L. Lusuardi, S. Ferretti, O. F. Cavdar, G. Ortner, S. Sultan, S. Choong, S. Micali, I. Saltirov, A. Sezer, C. Netsch, E. de Lorenzis, O. O. Cakir, G. Zeng, A. S. Gozen, G. Bianchi, B. Jurkiewicz, T. Knoll, J. Rassweiler, K. Ahmed, K. Sarica

**Affiliations:** 1 Department of Urology, Necmettin Erbakan University Meram School of Medicine, Konya, Turkey; 2 Department of Urology, University General Hospital of Heraklion, Athens, Greece; 3 Department of Urology, Trakya University School of Medicine Hospital, Edirne, Turkey; 4 Ordensklinikum Linz, Barmherzige Scwestern Hospital, Linz, Austria; 5 Department of Urology, University of Washington, Seattle Children’s Hospital, Seattle, WA USA; 6 Istanbul Medipol University, Istanbul, Turkey; 7 Preeti Urology Hospital, Hyderabad, Telangana India; 8 Hospital Universitario La Paz, Madrid, Spain; 9 Department of Urology, Tabba Kidney Institute, Karachi, Pakistan; 10 Department of Urology, Technical University Munich, Munich, Germany; 11 Urology Department, Fundación Puigvert, Universidad Autónoma de Barcelona, Barcelona, Spain; 12 Pediatric Surgery and Urology Clinic CMKP in Dziekanów Leśny, Dziekanów Leśny, Poland; 13 Department of Urology, Paracelsus Medical University Salzburg University Hospital, Urology, Salzburg, Austria; 14 Department of Urology, University of Modena and Reggio Emilia, Modena, Italy; 15 Department of Urology, General Hospital Hall I.T, Tirol, Austria; 16 Department of Urology, Menoufia University Hospitals, Shebeen El Kom, Egypt; 17 Institute of Urology, University College Hospital, London, UK; 18 Department of Urology and Nephrology, Military Medical Academy, Sofia, Bulgaria; 19 Pediatric Urology Clinic, Konya City Hospital, Konya, Turkey; 20 Asklepios Klinik BarmbekAbteilung Für Urologie, Hamburg, Germany; 21 Department of Urology, Fondazione IRCCS Ca’ Granda Ospedale Maggiore Policlinico, Milan, Italy; 22 King’s College London, Guy’s and St. Thomas’ NHS Foundation Trust, King’s Health Partners, London, UK; 23 Department of Urology and Guangdong Key Laboratory of Urology, The First Affiliated Hospital of Guangzhou Medical University, Guangzhou, China; 24 Department of Urology, Medius Clinic, Ostfildern, Germany; 25 Klinikum Sindelfingen-Boeblingen, Sindelfingen, Germany; 26 Department of Urology and Andrology, Danube Private University, Krems, Austria; 27 Sheikh Khalifa Medical City, Abu Dhabi, UAE; 28 Khalifa University, Abu Dhabi, UAE; 29 Sancaktepe Sehit Prof. Dr. Ilhan Varank Research and Training Hospital, Istanbul, Turkey; 30 Department of Urology, Biruni University Medical School, Istanbul, Turkey

**Correction to: World Journal of Urology (2024) 42:473**

10.1007/s00345-024-05161-4

In this article, the second affiliation of the last author K. Sarica was missed. It should be as given below:

Department of Urology, Biruni University Medical School, Istanbul, Turkey

Now, the original article has been corrected.

