# Supplementary material for: Consensus statement addressing controversies and guidelines on pediatric urolithiasis
Source: World J Urol. 2024 Aug 7;42(1):473. doi: 10.1007/s00345-024-05161-4 (PMC11306500; doi:10.1007/s00345-024-05161-4)
Supplement: Supplementary file 1 — Supplementary file1 (DOCX 17 KB) [file 345_2024_5161_MOESM1_ESM.docx]

***Pediatric Urolithiasis Survey***

1. Name Surname

*Type your answer here*

1. Affiliation/Institution

*Type your answer here*

1. Position/Title

*Type your answer here*

1. Years of Experience
2. *0-5 years*
3. *6-10 years*
4. *11-15 years*
5. *16+ years*
6. Please identify and briefly describe any controversial issues or points you believe exist within the field of pediatric urolithiasis. You can select multiple options and provide additional details for each choice:
7. *Lack of consensus on diagnosis criteria*
8. *Disagreement on optimal treatment approaches*
9. *Variability in follow-up protocols*
10. *Other (please explain)*
11. What do you believe are the primary factors contributing to these controversies? You can select multiple options and provide additional details for each choice:
12. *Differing interpretations of research findings*
13. *Lack of standardized guidelines*
14. *Variability in patient populations*
15. *Insufficient or Lack of evidence*
16. *Other (please explain)*
17. How do you perceive the current practices and recommendations regarding the management and treatment of pediatric urolithiasis? Please provide additional details:

*Type your answer here*

1. In your opinion, what specific areas within pediatric urolithiasis require further research to address these controversies and improve patient outcomes? Please provide additional details:

*Type your answer here*

1. Are there any additional questions you would like to circulate in the second round of this project before deciding on the themes?

*Type your answer here*

1. Additional Comments

*Type your answer here*
