# Supplementary material for: Consensus statement addressing controversies and guidelines on pediatric urolithiasis
Source: World J Urol. 2024 Aug 7;42(1):473. doi: 10.1007/s00345-024-05161-4 (PMC11306500; doi:10.1007/s00345-024-05161-4)
Supplement: Supplementary file 2 — Supplementary file2 (DOCX 87 KB) [file 345_2024_5161_MOESM2_ESM.docx]

| **Pediatric Urolithiasis Survey Step II** | **Average Point** | **Agreement (%)** |
| --- | --- | --- |
| 1. Diagnosis Criteria and Protocols  *There is a critical need for consensus on diagnosis criteria and protocols for pediatric urolithiasis.* | 4.6 | 92 |
| 2. Diagnosis Criteria and Protocols  *Standardizing diagnostic tools and defining clear intervention thresholds is essential for the field.* | 4.6 | 100 |
| 3. Treatment Approaches  *It is important to clarify and compare treatment modalities for pediatric urolithiasis, ensuring they are tailored to patient-specific factors.* | 4.6 | 92 |
| 4. Follow-up Protocols  *The standardization of follow-up protocols is necessary to establish best practices for monitoring patients after diagnosis or treatment of pediatric urolithiasis.* | 4.8 | 100 |
| 5. Evidence and Research Gaps  *Addressing the current lack of pediatric-specific evidence and research is urgent to guide clinical decisions and improve patient outcomes in pediatric urolithiasis.* | 4.6 | 100 |
| 6. Guidelines  *There is a need to review and harmonize existing guidelines from various bodies to identify overlaps and gaps in pediatric urolithiasis care.* | 4.6 | 92 |
| 7. Guidelines  *Creating integrated, evidence-based recommendations for pediatric urolithiasis is a high priority.* | 4.6 | 100 |
| 8. Training and Expertise  *Enhancing the quality of care in pediatric endourology requires emphasizing specialized training.* | 4.6 | 92 |
| 9. Technological Advances  *Research into new technologies and methods, including advanced imaging and endoscopic equipment, is crucial for improving treatment in pediatric urolithiasis.* | 4.5 | 88 |
| 10. Patient-Centric Considerations  *Pediatric urolithiasis care must be tailored to the diverse needs of patients, considering their unique physiological and socioeconomic factors.* | 4.6 | 96 |
| 11. Collaborative Efforts  *Encouraging collaborative research and the formation of coalitions among pediatric urologists is important for standardizing care practices in pediatric urolithiasis.* | 4.5 | 92 |
| 12. Do you agree with the content and priorities outlined in the table? If not, please specify which aspects you disagree with or believe should be amended. |  | 100 |
| 13. Diagnostic Approach  *The comparison between guidelines on the diagnostic approach (USG, KUB, CT) for pediatric urolithiasis was thorough and highlighted important methodological differences.* | 4.1 | 76 |
| 14. Stone Composition  *Varied focus on stone composition suggests the need for more unified guidelines.* | 4.5 | 92 |
| 15. Management for Small Stones  *The guidelines' diversity in managing small stones indicates room for alignment.* | 4.3 | 92 |
| 16. Shockwave Lithotripsy (SWL)  *Recommendations for SWL show differences that may benefit from further clarification.* | 4.3 | 84 |
| 17. Ureteroscopy (URS)  *Discrepancies in URS guidance across guidelines merit closer examination.* | 4.4 | 92 |
| 18. Percutaneous Nephrolithotomy (PCNL)  *PCNL recommendations reflect differences that could be harmonized.* | 4.4 | 92 |
| 19. Metabolic Evaluation  *Inconsistencies in metabolic evaluation guidance are important to address.* | 4.6 | 96 |
| 20. Minimally Invasive Techniques  *Diverse opinions on minimally invasive techniques point to a need for discussion.* | 4.4 | 96 |
| 21. Age-tailored Management  *The variation in age-tailored management suggests a need for specific pediatric protocols.* | 4.4 | 84 |
| 22. Overall Assessment  *The comparative analysis identifies important guideline discrepancies worth exploring further.* | 4.5 | 96 |
| 23. Do you agree with the content and priorities outlined in the table? If not, please specify which aspects you disagree with or believe should be amended. |  | 100 |
| 24. Diagnostic Criteria and Tools  *Conducting research to unify diagnostic criteria and develop pediatric-specific diagnostic tools is a critical priority.* | 4.5 | 100 |
| *25.* Treatment Modalities and Approaches  *Investigating optimal treatment methods, tailored to variables like stone size, location, and individual patient factors, is crucial.* | 4.6 | 92 |
| 26. Treatment Modalities and Approaches  *Research into the use of miniaturized scopes and the impact of emerging technologies on treatment is necessary.* | 4.4 | 88 |
| 27. Monitoring and Follow-up Care  *Standardizing follow-up protocols and reducing radiation exposure during imaging are key research priorities.* | 4.8 | 100 |
| 28. Monitoring and Follow-up Care  *Developing long-term monitoring strategies and managing residual fragments are crucial for preventing recurrence.* | 4.6 | 96 |
| 29. Evidence and Guidelines  *Addressing the lack of pediatric-specific evidence to develop more standardized guidelines is needed.* | 4.7 | 100 |
| 30. Socioeconomic and Institutional Factors  *Examining the influence of socioeconomic and institutional factors on the management of pediatric urolithiasis is important.* | 4.4 | 88 |
| 31. Professional Training and Specialization  *Specialized training and the need for dedicated pediatric urologists should be a significant focus of research.* | 4.6 | 88 |
| 32. Collaborative Efforts and Global Perspectives  *Working towards consensus guidelines and global standardization is a priority that encompasses collaborative research, inclusivity, and assessing the environmental impact of new disposables.* | 4.5 | 92 |
| 33. Treatment Equipment and Innovation  *Advancing research into the miniaturization of surgical instruments and suction devices, the development of new disposable materials, and the application of innovative technologies such as AI is essential.* | 4.4 | 84 |
| 34. Long-Term Impact and Comparative Studies  *Studying the long-term effects of SWL, the thermal effects of lasers, and comparing different treatment modalities through multicentric RCTs is vital.* | 4.6 | 92 |
| 35. Do you agree with the content and priorities outlined in the table? If not, please specify which aspects you disagree with or believe should be amended. |  | 96 |
| 36. Survey Preparation and Distribution  *The initial 10-question survey was appropriately designed to capture a comprehensive understanding of expert opinions on pediatric urolithiasis.* | 4.6 | 100 |
| 37. Data Collection  *The process used for collecting the surveys was systematic and reliable, ensuring that all expert responses were gathered and aggregated correctly.* | 4.7 | 100 |
| 38. Data Analysis and Theme Identification  *The analysis of the aggregated data was conducted rigorously to identify key issues in pediatric urolithiasis accurately.* | 4.6 | 100 |
| 39. Data Analysis and Theme Identification  *The methodology used was effective in distilling main themes that reflect the current state of pediatric urolithiasis management.* | 4.6 | 100 |
| 40. Guideline Review and Comparison  *The review process of the EAU, AUA, and IAU guidelines was robust and critical in finding responses to the controversial issues identified in the study.* | 4.6 | 96 |
| 41. Guideline Review and Comparison  *The comparative analysis of the guidelines was comprehensive and provided a detailed assessment of discrepancies and areas of consensus.* | 4.6 | 96 |
| 42. Expert Panel Deliberation  *The methodology for conducting the second survey among experts promoted a constructive dialogue aimed at reaching a consensus on controversial issues.* | 4.8 | 100 |
| 43. Expert Panel Deliberation  *The process of integrating insights from the guideline review into expert panel deliberations was transparent and contributed meaningfully to the discussions.* | 4.7 | 96 |
| 44. Consensus Statement  *The approach taken to draft the consensus statement effectively addressed the identified issues and integrated expert panel insights.* | 4.5 | 100 |
| 45. Consensus Statement  *The final consensus statement is a product of a collaborative effort that accurately reflects the expert consensus on the methodology used in the study.* | 4.7 | 96 |
| 46. Do you agree with the content and priorities outlined in the table? If not, please specify which aspects you disagree with or believe should be amended. |  | 100 |

| **Years of experience according to Pediatric Urolithiasis Survey Step I** | **Number of respondents** |
| --- | --- |
| 16 + years  11-15 years  6-10 years  0-5 years | 20  2  2  6 |

Figure 1. The results of related questions about “Themes identified from initial survey” from Pediatric Urolithiasis Survey Step 2

Figure 2. The results of related questions about “Guidelines Comparison” from Pediatric Urolithiasis Survey Step 2

Figure 3. The results of related questions about “Research Priorities in Pediatric Urolithiasis” from Pediatric Urolithiasis Survey Step 2

Figure 4. The results of related questions about “Consensus Study Development Methodology” from Pediatric Urolithiasis Survey Step 2
